# Local Tumour Control Following Microwave Ablation: Protocol for the Prospective Observational CIEMAR Study

**DOI:** 10.1007/s00270-023-03573-0

**Published:** 2023-10-26

**Authors:** Philippe L. Pereira, Reto Bale, Åsmund Avdem Fretland, S. Nahum Goldberg, Thomas Helmberger, Martijn R. Meijerink, Franco Orsi, Stefan Stättner, Thomas Vogl, Anna Kafkoula, Niels de Jong, Bleranda Zeka, Thierry de Baère

**Affiliations:** 1 Center of Radiology, Minimally Invasive Therapies and Nuclear Medicine, SLK-Kliniken GmbH, Heilbronn, Germany; 2 Academic Hospital University Heidelberg, Heidelberg, Germany; 3 Eberhards-University Tübingen, Tübingen, Germany; 4 Danube Private University Krems, Krems a/d Donau, Austria; 5 Department of Radiology, Section of Interventional Oncology-Microinvasive Therapy (SIP), Medical University of Innsbruck, Anichstr. 35, 6020 Innsbruck, Austria; 6 The Intervention Centre, Oslo University Hospital, Oslo, Norway; 7 Department of Hepato-Pancreatic-Biliary Surgery, Oslo University Hospital, Oslo, Norway; 8 Department of Radiology, Hadassah Hebrew University Medical Center, Jerusalem, Israel; 9 Department of Radiology, Neuroradiology and Minimal-Invasive Therapy, Klinikum Bogenhausen, Englschalkinger Str. 77, 81925 Munich, Germany; 10 Department of Radiology and Nuclear Medicine, Amsterdam University Medical Centers, Location VUmc De Boelelaan 1117, 1081 HV Amsterdam, The Netherlands; 11 Divisione Di Radiologia Interventistica, Istituto Europeo Di Oncologia, Istituto Di Ricovero E Cura a Carattere Scientifico (IRCCS), Milan, Italy; 12 Department of General, Visceral and Vascular Surgery, SKG Kliniken Vöcklabruck and Gmunden, Vöcklabruck, Gmunden, Austria; 13 Department of Radiology, University Hospital Frankfurt, Frankfurt, Germany; 14 Clinical Research, Cardiovascular and Interventional Radiological Society of Europe, Neutorgasse 9, 1010 Vienna, Austria; 15 Departement d’Anesthésie, de Chirurgie, Et de Radiologie Interventionnelle, Gustave Roussy, 102 Rue Edourad Vaillant, Villejuif, France; 16 Université Paris-Saclay, UFR Médecine Le Kremlin-Bicêtre, Le Kremlin Bicêtre, France; 17 Centre d’Investigation Clinique BIOTHERIS, INSERM CIC1428, 102 Rue Edourad Vaillant, Villejuif, France

**Keywords:** Colorectal liver metastases, Observational study, Registries, Liver, Microwave ablation

## Abstract

**Purpose:**

Microwave ablation (MWA) is a treatment modality for colorectal liver metastases (CRLM). While potentially curative, more information is needed on factors that contribute to long-term local tumour control. The prospective multicentre observational study CIRSE Emprint Microwave Ablation Registry aims to prospectively collect real-world technical data and clinical outcomes on patients treated with MWA in CRLM.

**Methods:**

Eligible patients are adults with up to 9 local treatment naïve CRLM of ≤ 3 cm completely treatable with either MWA alone or MWA with resection and/or radiotherapy within 8 weeks. Data are collected, at baseline, every 3 months until 12 months, and thereafter every 6 months until the end of the study. The primary outcome measure is local tumour control. Secondary outcome measures are overall survival, (hepatic-) disease-free survival, time-to-progression untreatable by ablation, systemic therapy vacation, safety, and quality of life. Covariates related to the primary outcome measure will be assessed using a stratified log-rank test and an univariable Cox proportional hazard regression. A sample size of 500 patients with 750 lesions produces a two-sided 95% confidence interval with a precision equal to 0.057.

**Results:**

Between September 2019 and December 2022, 500 patients have been enrolled with at least 976 treated tumours.

**Conclusion:**

The prospective observational CIEMAR study will provide valuable insights into the real-world use of MWA, helping in the future patient selection and clarifying factors that may contribute to long-term local tumour control.

*Trial Registration*: NCT03775980.

**Graphical Abstract:**

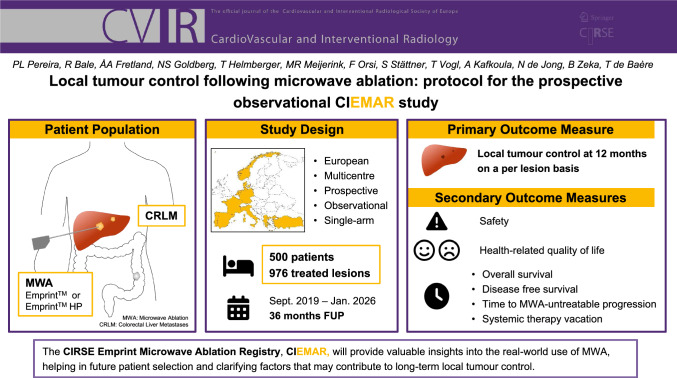

**Supplementary Information:**

The online version contains supplementary material available at 10.1007/s00270-023-03573-0.

## Introduction

Thermal ablation is a potentially curative treatment option for patients with colorectal liver metastases (CRLM) [1, 2]. In oligometastatic disease, characterised by one to five metastatic lesions in up to two metastatic sites that can be treated safely by local treatment, surgery remains the standard procedure, achieving a 5-year disease-free survival rate of approximately 20% [3, 4] and a median 5-year survival of 38% [5]. Long-term survival or cure is achievable in 20–45% of the patients who undergo complete A0 thermal ablation [6, 7].

In unresectable and more advanced disease, thermal ablation may be part of a multimodal approach in combination with systemic treatment. Common systemic therapies in early lines are a combination of irinotecan and/or oxaliplatin with 5-fluorouracil/leucovorin together with biologicals such as cetuximab or panitumumab (anti-epidermal growth factor receptor) and bevacizumab or aflibercept (anti-vascular endothelial growth factor) [2]. The randomised phase 2 CLOCC trial has shown that the addition of radiofrequency ablation (RFA)—with or without resection—to systemic treatment prolongs overall survival (OS) significantly (hazard ratio [HR] 0.58, 95% confidence interval [CI] 0.38–0.88, *p* = 0.01) in patients with primarily unresectable CRLM compared with systemic treatments alone [8].

Despite the stronger evidence base for RFA, microwave ablation (MWA) has shown similar effectiveness outcomes [6, 9–12] while adding the benefit of being less susceptible to the heat-sinking effect, which leads to more predictable and larger ablation zones compared with RFA [13–15]. Unlike RFA, MWA is a newer technique and clinical studies on MWA lack systematically collected prospective long-term follow-up data, which limits the understanding of the potential long-term benefits of this ablation modality [16]. The currently ongoing randomised studies COLLISION (NCT03088150), COLLISION-XL (NCT04081168) and NEW-COMET (NCT05129787) also aim to improve these shortcomings [17].

Awaiting the results of these trials, the Cardiovascular and Interventional Radiological Society of Europe (CIRSE) initiated the multicentre prospective observational study CIRSE Emprint™ Microwave Ablation Registry (CIEMAR, NCT03775980). CIEMAR aims to study the long-term effectiveness of MWA in CRLM in the real-world clinical setting, including important parameters such as health-related quality of life (HRQOL), which provides a “real-world” counterpart to the COLLISION trial, to form a substantial evidence base for the use of MWA in CRLM. The study is sponsored and conducted by CIRSE.

## Methods

### Study design

CIEMAR is a prospective, single-arm, multi-centre observational study observing the use of MWA in Europe as performed in routine clinical practice for the treatment of patients suffering from colorectal liver metastases.

The study is governed by a multidisciplinary scientific steering committee including interventional radiologists and liver surgeons. This study was performed in accordance with the declaration of Helsinki and good clinical practice guidelines and was approved by the local ethics committees of participating centres. All patients were required to sign an informed consent for upon study enrolment.

### Site selection and patient enrolment

Sites using Emprint™ or Emprint HP™ as the standard of care for CRLM and meeting a minimum amount of experience (a minimum of 20 ablations of liver tumours using any thermal ablation method per year within the last 4 years) were identified and invited to participate in the study. Centres from 16 European nations (Belgium, Croatia, Denmark, France, Germany, Greece, Italy, the Netherlands, Northern Macedonia, Norway, Portugal, Spain, Sweden, Switzerland, Turkey, and the United Kingdom) were asked to participate.

Patient inclusion and exclusion criteria are displayed in Table 1. Site enrolment started in October 2018 and ended in November 2022. Patient enrolment started in September 2019 and was completed in January 2023.

**Table 1:** Patient inclusion and exclusion criteria

Inclusion criteria
18 years old or older
Proven colorectal liver metastases (either histologically or diagnosed by imaging in a patient with known colorectal cancer)
Patient referred to MWA by a multidisciplinary tumour board
Treated with the Emprint or Emprint HP Microwave ablation system
Intention to completely treat (ablation, resection, Stereotactic body radiation therapy [SBRT]) all visible disease within 8 weeks
Max. number of 9 liver lesions
All liver lesions local treatment-naive
Max. diameter of the largest liver lesion treated does not exceed 3 cm

Exclusion criteria
Life expectancy less than 6 months (palliative treatment)
Extrahepatic metastases with the exception of a maximum of 5 lung nodules
Ongoing infection (viral/bacterial)
Patients receiving simultaneous bowel surgery and microwave ablation
Patients receiving simultaneous irreversible electroporation, RFA, SBRT, Cryoablation, high-intensity focused ultrasound, or other local treatment than resection
Pregnancy
Patients with liver metastases that cannot be completely and safely treated
Active cancers other than non-resected primary colon cancer
Advanced liver disease or evidence of liver insufficiency

### Data collection and quality control

**Fig. 1:**
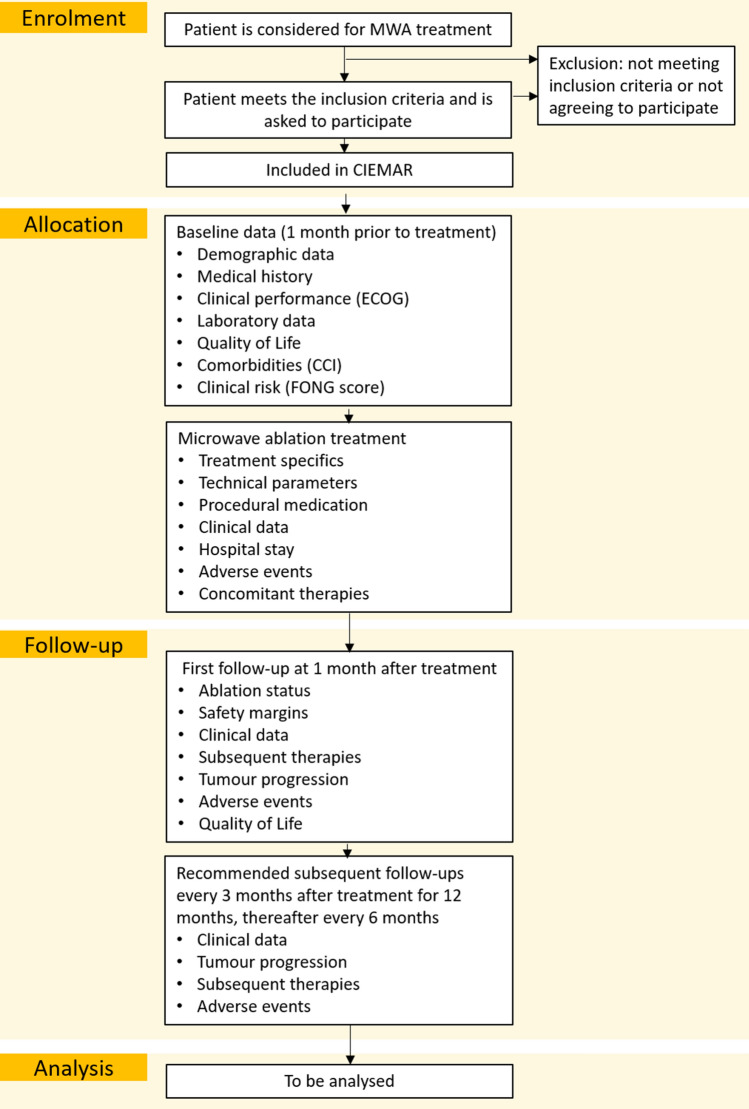
Flowchart detailing the data collected at each stage of the enrolment. ECOG: Eastern Cooperative Oncology Group; CCI: Charlson Comorbidity Index

Patients enter the study by accepting participation and signing the informed consent form. Baseline data are collected one month prior to the first treatment (see Fig. 1). Additionally, the relevant data to calculate the baseline Charlson Comorbidity Index (CCI) and the clinical risk score (Fong Score) are collected [18, 19]. After each treatment, technical parameters are collected on how the treatment is performed, whether simultaneous resection of other lesions is performed, and whether concomitant systemic therapy is received. Follow-up data are collected 1 month after the treatment, then every 3 months for the first 12 months and then every 6 months until the study exit or study end, 36 months after the 500th patient has been included. Data for safety and adverse events are collected throughout the whole study. HRQOL data are collected before the first treatment and at the first follow-up.

Data validity, completeness, consistency, and uniformity are warranted by case report forms with programmed constraints on data types, ranges, and mandatory data fields. Furthermore, a regular remote monitoring scheme is employed to verify the data quality, inclusion of patients, and follow-ups. No on-site monitoring or source data verification is performed due to resource limitations. Remote monitoring is performed quarterly. Activities include monitoring calls with the sites, sending query reports and monitoring call reports in which all necessary action points are documented. Data are monitored regularly, and queries are raised via the electronic data capturing system. The non-interventional nature of the study means that the exact dates of the procedures and follow-ups are determined by the medical team.

The electronic data collection system was developed and supported by OpenClinica (LLC, Waltham, MA, USA).

### Data sets

For the main results analysis, a full analysis data set composed of all eligible patients who were enrolled in the registry and underwent MWA treatment will be used. Patients for whom the amount of data collected is too scarce to warrant meaningful analysis might be assessed for exclusion. The cut-offs for data scarcity will be evaluated during the final analysis, and the number of excluded patients as well as the reason for exclusion will be reported. Other data sets (e.g. to explore country or centre effects) might be created during the final analysis.

### Primary outcome measure

The primary outcome measure is the local control rate for all ablated tumours at 1 year (defined as fully ablated with no evidence of disease within the ablation zone at 12 months + / − 45 days after the last ablation of the respective tumour) as assessed by the investigatorsʼ routine protocols (Table 2).

**Table 2:** Primary and secondary outcome measures

	Outcome measure	Measured according to	Definition
*Primary outcome measure*			
Effectiveness	Local tumour control at 12 months on a per lesion basis	Investigator assessment	Tumour is considered to be fully ablated with no evidence of disease within the ablation zone at 12 months + / − 45 days after the last ablation
*Secondary outcome measures*			
Safety	Adverse events	CTCAE V5.0	
Effectiveness	Overall survival	Investigator assessment	Time between the first treatment and the time of death of any cause
	Overall disease-free survival	Investigator assessment	Time between the first treatment and the time of any progression, new tumours, or death
	Hepatic disease-free survival	Investigator assessment	Time between the first treatment and the time of any progression of hepatic tumours, new hepatic tumours or death
	Time to untreatable progression by thermal ablation	Investigator assessment	Time between the first treatment and the time of progression of a treated tumour that has been determined as not treatable by thermal ablation
	Systemic cancer therapy vacation	Investigator assessment	Time between the first treatment and the start of systemic cancer therapy
	Quality of Life	EORTC QLQ-C30	

### Secondary outcome measures

#### Safety

Safety will be descriptively analysed based on reported adverse events (AEs) and abnormal laboratory values (biologic AEs) recorded during patient follow-up. Reported AEs and biologic AEs will be graded according to the Common Terminology Criteria for Adverse Events (CTCAE), version 5.0 and reported by the PIs. They will be summarised as the total number of AEs and the percentage of patients with at least one AE for any grade, grades 3–4, grade 5 and related or likely to be related will be given.

#### Time-to-event

The first MWA treatment will be the starting point for the per-patient time-to-event analyses, and the first MWA treatment of the respective lesion for per-lesion time-to-event analyses. OS is defined as the time of death of any cause, for DFS as the time of any progression, new tumours, or death, for HDFS as the time of any progression of liver tumours, new tumours in the liver or death and for time to progression untreatable by thermal ablation as the time of progression of a treated tumour that has been determined as not treatable by thermal ablation according to the investigator. Systemic cancer therapy vacation is defined as the time between the first MWA treatment and the start of systemic cancer therapy. In these analyses, subjects lost to follow-up will be censored at the last recorded communication with the patient.

#### Patient-reported quality of life

Health-related quality of life data will be analysed using version 3 of the *European Organisation for Research and Treatment of Cancer* (EORTC) QLQ-C30 Scoring Manual (2001) in all dimensions. Results will be presented for the global health status/QoL score, functional and symptom dimensions, and for individual items.

### Statistical analysis

#### Sample size calculation

As the total number of available lesions will depend on how many lesions per patient are treated, several scenarios were used to calculate the required sample size to achieve sufficient precision for analysis. Based on previously published data [11, 16], local tumour control rates of 60%, 70%, and 80% for 1, 1.5, and 2 tumours per patient were chosen as possible scenarios. Using the resulting sample size, the expected precisions were calculated using the two-sided 95% confidence interval according to the Wilson score method [20, 21].

A sample size of 500 patients with a median of 1.5 tumours per patient produces a two-sided 95% confidence interval with a precision equal to 0.057 when the sample proportion is 0.8.

#### Outcome measures

The median time to event and 95% confidence interval will be calculated using the Kaplan–Meier estimator. Results will be illustrated graphically using Kaplan–Meier plots and the impact of different covariates will be assessed using a stratified log-rank test and an univariable Cox proportional hazard regression. The pre-defined list of covariates can be found in Supplement 1. Covariates that have at least a 10–90% ratio and no more than 10% missing data will be used. Covariates with *p* values of < 0.1 will be included in a multivariable Cox proportional hazard regression model. Backwards elimination will be used for model building. The criteria for retaining variables in the model based on statistical significance (*p* < 0.05) and model improvement. The Cox proportional hazard assumptions will be verified and covariates that do not satisfy those assumptions will be flagged.

Quality of life outcomes will be presented by absolute values and change from baseline using summary statistics. Summaries will be broken down into subgroups and covariate analyses may be performed using analysis of covariance (ANCOVA) depending on the number of available questionnaires at the final analysis.

All statistical tests will be based on a significance level of 5%, and estimates will be presented using two-sided 95% confidence intervals. As it is not planned to test any statistical hypotheses in a confirmatory sense, statistical testing will have to be interpreted in the perspective of the study.

#### Missing data

Missing observations will be presented as a percentage of the total. For the assessment of the primary outcome measure, a drop-out is defined as a lesion not followed up to the 12 months + / − 45 days mark. In the case of the death of a patient before the 12 months assessment, only lesions that had progressed will be regarded as not having achieved tumour control at 12 months and lesions where progression could not be determined before the death of the patient will be regarded as a drop-out. A sensitivity analysis will be done to assess potential bias due to drop-outs before the 1-year assessment. Depending on the differences between the primary analysis and the sensitivity analysis, a tipping-point analysis may be performed.

The statistical analysis will be done using R Studio version 4.4.

## Current status of the study

Between October 2018 and June 2022, 100 hospitals that met the selection criteria were invited, of which 36 were activated (by means of training, access to the database, and eligibility to include patients). As of January 2023, 500 patients have been enrolled with 976 treated tumours, achieving the minimum sample size of 976 lesions for a precision between 0.04 and 0.06. Follow-up data will be collected until January 2026. The final clinical outcome results are expected to be published by 2027.

## Discussion

CIEMAR is a Europe-wide, prospective multicentre observational study of patients with colorectal liver metastases treated with microwave ablation as standard therapy. Its primary outcome measure is to evaluate local tumour control, with secondary outcome measures being overall survival, (hepatic) disease-free survival, time to progression untreatable by thermal ablation, systemic cancer therapy vacation, safety and quality of life.

The robust data of real-world, 1-year tumour control per lesion will be used to determine factors that impact the effectiveness of MWA and to identify unresectable lesions that may still be optimal for MWA, thus potentially improving the curative approach to CRLM. While the level of evidence of observational data is considered low compared to randomised controlled trials (RCTs), several comparative studies have shown that outcomes of RCTs and observational studies are comparable in terms of treatment effect and quality [22–24]. Nevertheless, a recent meta-analysis showed that the risk of bias was directly correlated with the effect estimate [25]. Therefore, the choice of a robust primary outcome measure, inclusion criteria, and prospective data collection are essential in reducing the risk of bias [26–28]. In this study, the choice of a primary outcome measure appropriate for localised treatments allows CIEMAR to precisely evaluate the extent of local tumour control with MWA and relate this to its secondary effectiveness outcome measures (26). Hence, CIEMAR will combine the strengths of the observational design with the localised effects of interventional oncological treatments and thus be able to avoid the pitfalls of observational studies that focus on more general outcome measures, such as OS or progression-free survival.

Initially, CIEMAR aimed to include 700 patients with 1050 lesions with a precision of 0.05. Unfortunately, due to the COVID-19 pandemic, some centres were understaffed or even closed, while in other cases, resources had to be re-allocated to cope with the influx of COVID-19 patients and cancer treatments, including ablation treatments, were postponed or aborted. This had a negative impact on patient enrolment. Nevertheless, the current sample size of a minimum of 803 lesions with a precision of 0.04–0.07 is statistically sufficient to evaluate the primary outcome measure.

### Limitations

The limitation of this study is that it has a single-arm observational design without a comparison group. Potentially unaccounted confounding factors may limit the interpretation of the data. Furthermore, as a single-device study using only the Emprint™ Microwave Ablation System and the new generation Emprint™ HP Microwave Ablation Systems, the outcomes may be limited in their generalisability to the numerous MWA devices existing on the market.

The observational design limits the ability to prescribe precise follow-up periods. While a 1-month follow-up after ablative treatment and a 3-month follow-up thereafter can be considered a standard of practice, missing data regarding quality-of-life questionnaires at this time interval can be expected. This will be mitigated with remote monitoring practices but needs to be considered when interpreting the outcomes. Additionally, the lack of on-site monitoring represents a limitation of the study. The data are provided and verified by the sites, but no source document verification is performed.

## Conclusion

CIEMAR is a large prospective multicentre study on the effectiveness of MWA in real-world clinical practice. Its outcomes will improve the understanding of factors that may contribute to local tumour control after MWA in CRLM. First results may be expected in 2027.

### Supplementary Information

Below is the link to the electronic supplementary material.

Supplementary file1 (DOCX 14 KB)

## Abbreviations

AE: Adverse event
CCI: Charlson Comorbidity Index
CI: Confidence interval
CIRSE: Cardiovascular and Interventional Radiological Society of Europe
CIEMAR: CIRSE Emprint™ Microwave Ablation Registry
CRLM: Colorectal liver metastases
CTCAE: Common terminology criteria for adverse events
DFS: Disease-free survival
EORTC: European Organisation for Research and Treatment of Cancer
HDFS: Hepatic disease-free survival
HRQOL: Health-related quality of life
HR: Hazard ratio
MWA: Microwave ablation
RFA: Radiofrequency ablation
OS: Overall survival
SBRT: Stereotactic body radiation therapy

## References

[1] Benson AB, Venook AP, Al-Hawary MM, Arain MA, Chen YJ, Ciombor KK (2021). Colon cancer, version 2.2021, NCCN clinical practice guidelines in oncology. J Natl Compr Canc Netw..

[2] Cervantes A, Adam R, Rosello S, Arnold D, Normanno N, Taieb J (2022). Metastatic colorectal cancer: ESMO Clinical Practice Guideline for diagnosis, treatment and follow-up. Ann Oncol..

[3] Choti MA, Sitzmann JV, Tiburi MF, Sumetchotimetha W, Rangsin R, Schulick RD (2002). Trends in long-term survival following liver resection for hepatic colorectal metastases. Ann Surg.

[4] Pawlik TM, Scoggins CR, Zorzi D, Abdalla EK, Andres A, Eng C (2005). Effect of surgical margin status on survival and site of recurrence after hepatic resection for colorectal metastases. Ann Surg..

[5] Kanas GP, Taylor A, Primrose JN, Langeberg WJ, Kelsh MA, Mowat FS (2012). Survival after liver resection in metastatic colorectal cancer: review and meta-analysis of prognostic factors. Clin Epidemiol.

[6] Shady W, Petre EN, Do KG, Gonen M, Yarmohammadi H, Brown KT (2018). Percutaneous microwave versus radiofrequency ablation of colorectal liver metastases: ablation with clear margins (A0) provides the best local tumor control. J Vasc Interv Radiol..

[7] Wong SL, Mangu PB, Choti MA, Crocenzi TS, Dodd GD, Dorfman GS (2010). american society of clinical oncology 2009 clinical evidence review on radiofrequency ablation of hepatic metastases from colorectal cancer. J Clin Oncol.

[8] Ruers T, Van Coevorden F, Punt CJ, Pierie JE, Borel-Rinkes I, Ledermann JA (2017). Local treatment of unresectable colorectal liver metastases: results of a randomized phase II trial. J Natl Cancer Inst..

[9] Izzo F, Granata V, Grassi R, Fusco R, Palaia R, Delrio P (2019). Radiofrequency ablation and microwave ablation in liver tumors: an update. Oncologist.

[10] Di Martino M, Rompianesi G, Mora-Guzman I, Martin-Perez E, Montalti R, Troisi RI (2020). Systematic review and meta-analysis of local ablative therapies for resectable colorectal liver metastases. Eur J Surg Oncol.

[11] Pathak S, Jones R, Tang JM, Parmar C, Fenwick S, Malik H (2011). Ablative therapies for colorectal liver metastases: a systematic review. Colorectal Dis.

[12] Radosevic A, Quesada R, Serlavos C, Sanchez J, Zugazaga A, Sierra A (2022). Microwave versus radiofrequency ablation for the treatment of liver malignancies: a randomized controlled phase 2 trial. Sci Rep.

[13] Vogl TJ, Farshid P, Naguib NN, Darvishi A, Bazrafshan B, Mbalisike E (2014). Thermal ablation of liver metastases from colorectal cancer: radiofrequency, microwave and laser ablation therapies. Radiol Med.

[14] Vogl TJ, Nour-Eldin NA, Hammerstingl RM, Panahi B, Naguib NNN (2017). Microwave ablation (MWA): basics, technique and results in primary and metastatic liver neoplasms—review article. Rofo.

[15] Brace CL (2009). Radiofrequency and microwave ablation of the liver, lung, kidney, and bone: what are the differences?. Curr Probl Diagn Radiol.

[16] Puijk RS, Dijkstra M, van den Bemd BAT, Ruarus AH, Nieuwenhuizen S, Geboers B (2022). Improved outcomes of thermal ablation for colorectal liver metastases: a 10-year analysis from the prospective Amsterdam CORE registry (AmCORE). Cardiovasc Intervent Radiol.

[17] Puijk RS, Ruarus AH, Vroomen L, van Tilborg A, Scheffer HJ, Nielsen K (2018). Colorectal liver metastases: surgery versus thermal ablation (COLLISION)—a phase III single-blind prospective randomized controlled trial. BMC Cancer.

[18] Charlson ME, Pompei P, Ales KL, MacKenzie CR (1987). A new method of classifying prognostic comorbidity in longitudinal studies: development and validation. J Chronic Dis.

[19] Fong Y, Fortner J, Sun RL, Brennan MF, Blumgart LH (1999). Clinical score for predicting recurrence after hepatic resection for metastatic colorectal cancer: analysis of 1001 consecutive cases. Ann Surg..

[20] Fleiss JL, Levin BA, Paik MC (2003). Statistical methods for rates and proportions.

[21] Newcombe RG (1998). Two-sided confidence intervals for the single proportion: comparison of seven methods. Stat Med.

[22] Benson K, Hartz AJ (2000). A comparison of observational studies and randomized, controlled trials. N Engl J Med.

[23] Concato J, Shah N, Horwitz RI (2000). Randomized, controlled trials, observational studies, and the hierarchy of research designs. N Engl J Med.

[24] Ioannidis JP, Haidich AB, Pappa M, Pantazis N, Kokori SI, Tektonidou MG (2001). Comparison of evidence of treatment effects in randomized and nonrandomized studies. JAMA.

[25] Di Bona D, Carlucci P, Spataro F, Paoletti G, Heffler E, Pulkanen J (2023). Comparison of evidence of treatment effects in randomized and nonrandomized studies on allergen immunotherapy. Clin Exp Allergy.

[26] Ahmed M (2014). Technology assessment committee of the society of interventional R. image-guided tumor ablation: standardization of terminology and reporting criteria–a 10-year update: supplement to the consensus document. J Vasc Interv Radiol..

[27] Franklin JM, Gebski V, Poston GJ, Sharma RA (2015). Clinical trials of interventional oncology-moving from efficacy to outcomes. Nat Rev Clin Oncol.

[28] Frieden TR (2017). Evidence for health decision making—beyond randomized. Controll Trials N Engl J Med.

