# Correction to: Local Tumour Control Following Microwave Ablation: Protocol for the Prospective Observational CIEMAR Study

**DOI:** 10.1007/s00270-023-03618-4

**Published:** 2023-11-27

**Authors:** Philippe L. Pereira, Reto Bale, Åsmund Avdem Fretland, S. Nahum Goldberg, Thomas Helmberger, Martijn R. Meijerink, Franco Orsi, Stefan Stättner, Thomas Vogl, Anna Kafkoula, Niels de Jong, Bleranda Zeka, Thierry de Baère

**Affiliations:** 1 Center of Radiology, Minimally Invasive Therapies and Nuclear Medicine, SLK-Kliniken GmbH, Heilbronn, Germany; 2 Academic Hospital University Heidelberg, Heidelberg, Germany; 3 Eberhards-University Tübingen, Tübingen, Germany; 4 Danube Private University Krems, Krems a/d Donau, Austria; 5 Department of Radiology, Section of Interventional Oncology-Microinvasive Therapy (SIP), Medical University of Innsbruck, Anichstr. 35, 6020 Innsbruck, Austria; 6 The Intervention Centre, Oslo University Hospital, Oslo, Norway; 7 Department of Hepato-Pancreatic-Biliary Surgery, Oslo University Hospital, Oslo, Norway; 8 Department of Radiology, Hadassah Hebrew University Medical Center, Jerusalem, Israel; 9 Department of Radiology, Neuroradiology and Minimal-Invasive Therapy, Klinikum Bogenhausen, Englschalkinger Str. 77, 81925 Munich, Germany; 10 Department of Radiology and Nuclear Medicine, Amsterdam University Medical Centers, Location VUmc De Boelelaan 1117, 1081 HV Amsterdam, The Netherlands; 11 Divisione Di Radiologia Interventistica, Istituto Europeo Di Oncologia, Istituto Di Ricovero E Cura a Carattere Scientifico (IRCCS), Milan, Italy; 12 Department of General, Visceral and Vascular Surgery, SKG Kliniken Vöcklabruck and Gmunden, Vöcklabruck, Gmunden, Austria; 13 Department of Radiology, University Hospital Frankfurt, Frankfurt, Germany; 14 Clinical Research, Cardiovascular and Interventional Radiological Society of Europe, Neutorgasse 9, 1010 Vienna, Austria; 15 Departement d’Anesthésie, de Chirurgie, Et de Radiologie Interventionnelle, Gustave Roussy, 102 Rue Edourad Vaillant, Villejuif, France; 16 Université Paris-Saclay, UFR Médecine Le Kremlin-Bicêtre, Le Kremlin Bicêtre, France; 17 Centre d’Investigation Clinique BIOTHERIS, INSERM CIC1428, 102 Rue Edourad Vaillant, Villejuif, France

**Correction to: Cardiovasc Intervent Radiol** 10.1007/s00270-023-03573-0

In the original article, the affiliation of the corresponding author Niels de Jong is incorrect:

Departement d’Anesthésie, de Chirurgie, Et de Radiologie Interventionnelle, Gustave Roussy, 102 Rue Edourad Vaillant, Villejuif, France

The correct affiliation of the corresponding author Niels de Jong is:

Clinical Research, Cardiovascular and Interventional Radiological Society of Europe, Neutorgasse 9, 1010 Vienna, Austria.

