# Supplementary material for: Local Tumour Control Following Microwave Ablation: Protocol for the Prospective Observational CIEMAR Study
Source: Cardiovasc Intervent Radiol. 2023 Oct 26;47(1):121–9. doi: 10.1007/s00270-023-03573-0 (PMC10770225; doi:10.1007/s00270-023-03573-0)
Supplement: Supplementary file 1 — Supplementary file1 (DOCX 14 KB) [file 270_2023_3573_MOESM1_ESM.docx]

**Supplement 1: Covariates for time to event end points**

| Covariate | Variables |
| --- | --- |
| Age (years) | <70, ≥70 |
| Sex | Male / female |
| Image modality used for baseline imaging | CT / PET / MRI |
| Eastern Cooperative Oncology Group (ECOG) performance status | 0 / 1 / 2 or higher |
| RAS mutation | Yes / no |
| BRAF (V600E) | Yes/no |
| MMR status | (MSS / pMMR or MSI/dMMR) |
| CEA value >20 ng/L | Yes / No |
| CRC diagnosis  o Time since CRC diagnosis  o Primary tumour location  o Primary tumour treatment  o Previous treatment procedures  o Completeness of surgery  o Adjuvant systemic therapy (after successful resection)  o TNM of primary tumour  Liver metastases  o Time since liver metastases diagnosis  o Total number of liver lesions at baseline  o Mean and Median lesion size  o Presence of metastases in the lung  o Number of lung metastases  o Treatments for lung metastases with curative intent  o Previous treatments for liver metastases  o Tumour status in the liver at the time of baseline | Categorical  Left / Right  Yes / No  Yes / No  Yes / No  Yes / No  TNM values  Categorical  1 / 2-5 / 6-9  Mean / Median  Yes / No  1 / 2 / 3-5  Yes / No  Yes / No  Partial response / Stable disease / Progressive disease |
| Charlson comorbidity index (CCI) | Mild (1-2) / Moderate (3-4) / Severe (≥5) |
| Fong score | Low risk (0-2) / High risk (3-5) |
